# Case Report: Novel Likely Pathogenic *ACTN2* Variant Causing Heterogeneous Phenotype in a Korean Family With Left Ventricular Non-compaction

**DOI:** 10.3389/fped.2021.609389

**Published:** 2021-03-30

**Authors:** Joonhong Park, Yong Gon Cho, Ha Wook Park, Jung Sun Cho

**Affiliations:** ^1^Department of Laboratory Medicine, Jeonbuk National University Medical School and Hospital, Jeonju, South Korea; ^2^Research Institute of Clinical Medicine of Jeonbuk National University-Biomedical Research Institute of Jeonbuk National University Hospital, Jeonju, South Korea; ^3^Department of Cardiology, College of Medicine, The Catholic University of Korea, Seoul, South Korea

**Keywords:** left ventricular non-compaction, heterogeneous phenotype, *ACTN2* variant, exome sequencing, cardiac magnetic resonance imaging

## Abstract

Left ventricular non-compaction (LVNC) is a very rare primary cardiomyopathy with a genetic etiology, resulting from the failure of myocardial development during embryogenesis, and it carries a high risk of left ventricular dysfunction, thromboembolic phenomenon, and malignant arrhythmias. Here, we report the first case of familial LVNC in Korea, caused by a novel *ACTN2* missense variant. We performed duo exome sequencing (ES) to examine the genome of the proband and his father. A 15-year-old boy was admitted for the evaluation of exertional dyspnea for 2 weeks. He was diagnosed with LVNC with a dilated cardiomyopathy phenotype [left ventricular end-diastolic dimension 60 mm, interventricular septal dimension 8.2 mm by transthoracic echocardiography (TTE)]. For the screening of familial cardiomyopathy, TTE and cardiac magnetic resonance imaging (cMRI) were performed, which revealed hypertrophic and isolated LVNC in the proband's father and sister, respectively. In particular, the cMRI revealed dense hypertrabeculation with focal aneurysmal changes in the apical septal wall in the proband's father. ES of the father–son duo identified a novel heterozygous c.668T>C variant of the *ACTN2* gene (NM_001103.3:c.668T>C, p.Leu223Pro; no rsID) as the candidate cause of autosomal dominant LVNC. Sanger sequencing confirmed this novel variant in the proband, his father, and sister, but not in the proband's grandmother. Even within families harboring the same variant, a variable risk of adverse outcomes is common. Therefore, familial screening for patients with LVNC associated with *ACTN2* variant should be performed for early detection of the LVNC phenotype associated with poor outcomes, such as dilated LVNC.

## Introduction

Left ventricular non-compaction (LVNC) is a rare cardiomyopathy due to the failure of myocardial development during embryogenesis, and it is characterized by a high risk of left ventricular dysfunction, malignant arrhythmias, and thromboembolic phenomenon (1, 2). The prevalence of LVNC has been reported as 3 to 4% among patients with heart failure (3). During development, most of the heart muscle is a sponge-like meshwork of interwoven myocardial fibers (2, 4). As normal development progresses, these trabeculated structures undergo significant compaction that transforms them from spongy to solid fibers, which is particularly apparent in the ventricles, particularly in the left ventricle. To date, this type of hereditary cardiomyopathy is not fully understood; however, it was classified as primary cardiomyopathy of genetic origin by the American Heart Association in 2006, as well as described as a distinct entity by MOGE(S) classification (5). The European Society of Cardiology categorizes it as an unclassified cardiomyopathy (6). The disease is caused by heterozygous mutations in different genes coding for the cardiac sarcomere; calcium handling; and other cardiomyopathy related genes, including *MYH7, MYBPC3, TPM1, ACTC1, TNNT2, LMNA, LDB3, TMP1, TNNI3*, and *ACTN2*, of which, the most commonly affected are the sarcomere genes *MYH7, MYBPC3*, and *TPM1* (7–9). Mutations in the genes encoding the Z-disc or calcium-handling proteins account for <1% of the cases (10). Among them, mutations in the *ACTN2* have been implicated in mild to moderate forms of hypertrophic and dilated cardiomyopathy (#612158) (11). However, in the case of LVNC with congenital heart disease, disturbance of the NOTCH signaling pathway seems to be a part of the final common pathway for this form of the disease (12, 13).

In this report, we describe a heterogeneous phenotype in familial LVNC with a novel *ACTN2* missense variant identified by exome sequencing (ES).

## Case Presentation

**Figure 1:**
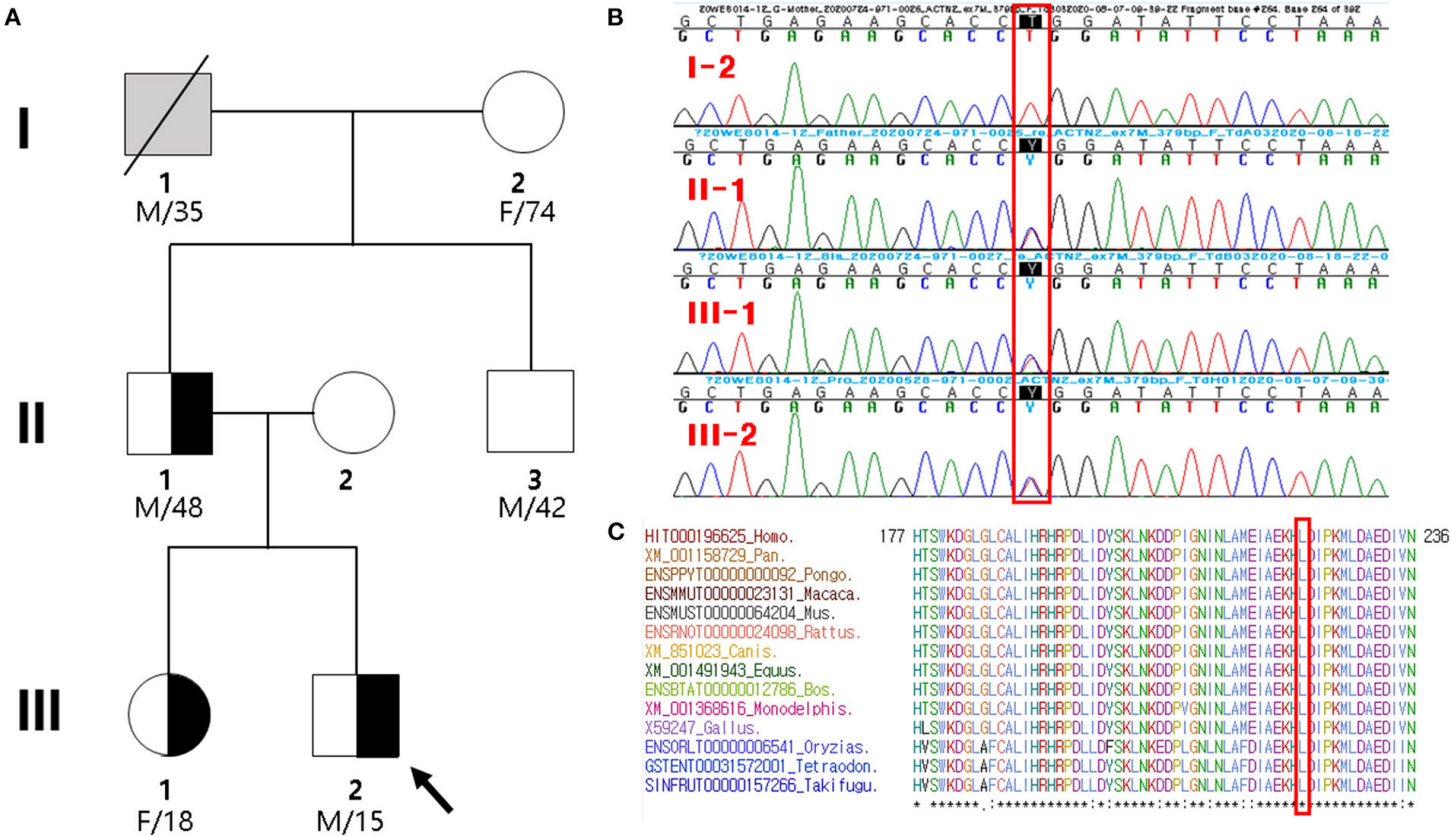
Pedigree analysis and sequencing results. **(A)** Pedigree of the proband (arrow) and his family members with heterozygous *ACTN2* variant. Gray symbol indicates that the presence of *ACTN2* variant was not determined. **(B)** Sanger sequencing confirmed a novel missense mutation (c.668T>C; p.Leu223Pro) in *ACTN2*, which was of paternal origin. It is highlighted in the red box. **(C)** Sequence alignment of the conserved cytoplasmic domain of the ACTN2 protein in multiple vertebrate species. Protein sequence of the Leu223Pro residue is highly conserved across the compared species. It is highlighted in the red box.

**Figure 2:**
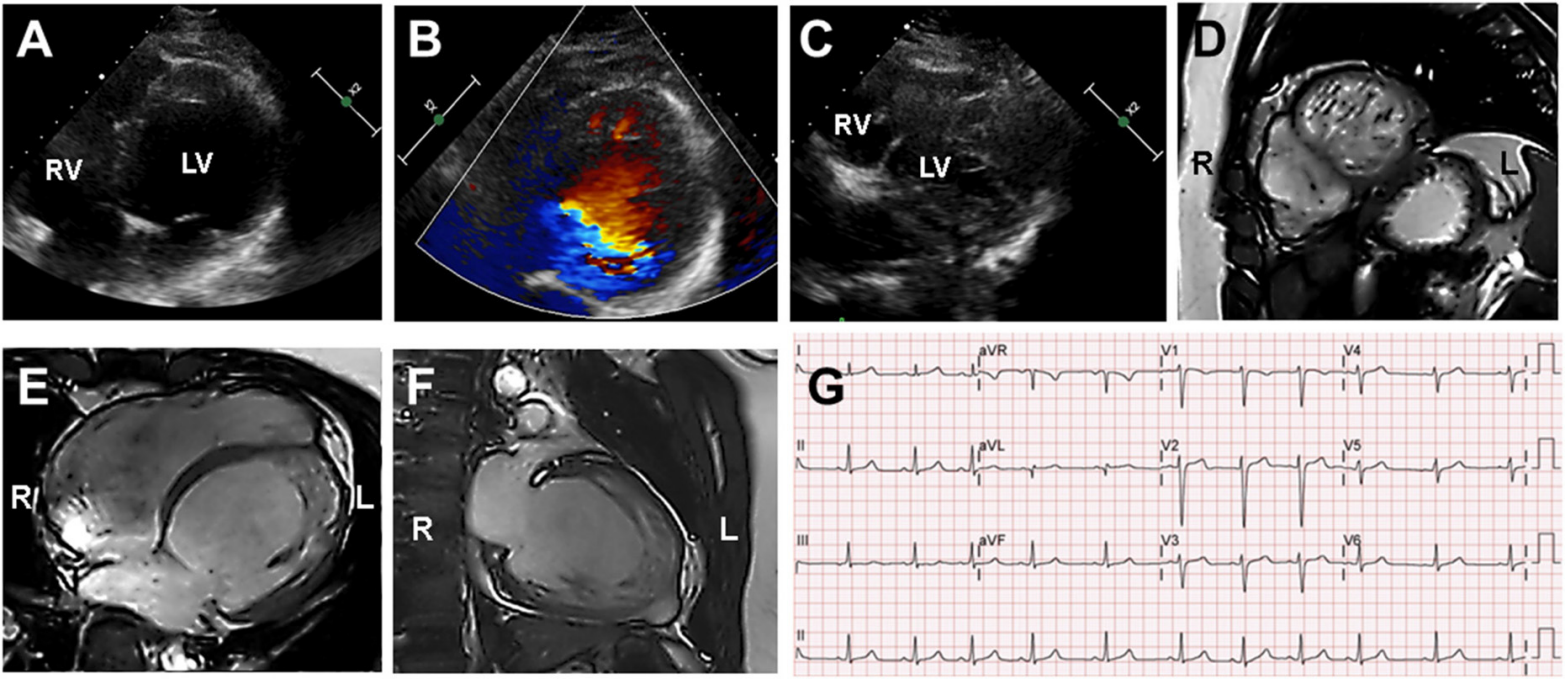
Echocardiography and cardiac magnetic resonance imaging (cMRI) in the proband (III-2). **(A–C)** Echocardiography and Color Doppler showed dilated left ventricle chamber and prominent trabeculation and flow within the deep intertrabecular recesses. **(D–F)** cMRI revealed that the non-compacted to compacted ratio ≥2 and severely dilated biventricular myocardium that was compatible with dilated LVNC. **(G)** Proband's electrocardiogram demonstrated sinus arrhythmia and non-specific intraventricular conduction delay.

**Figure 3:**
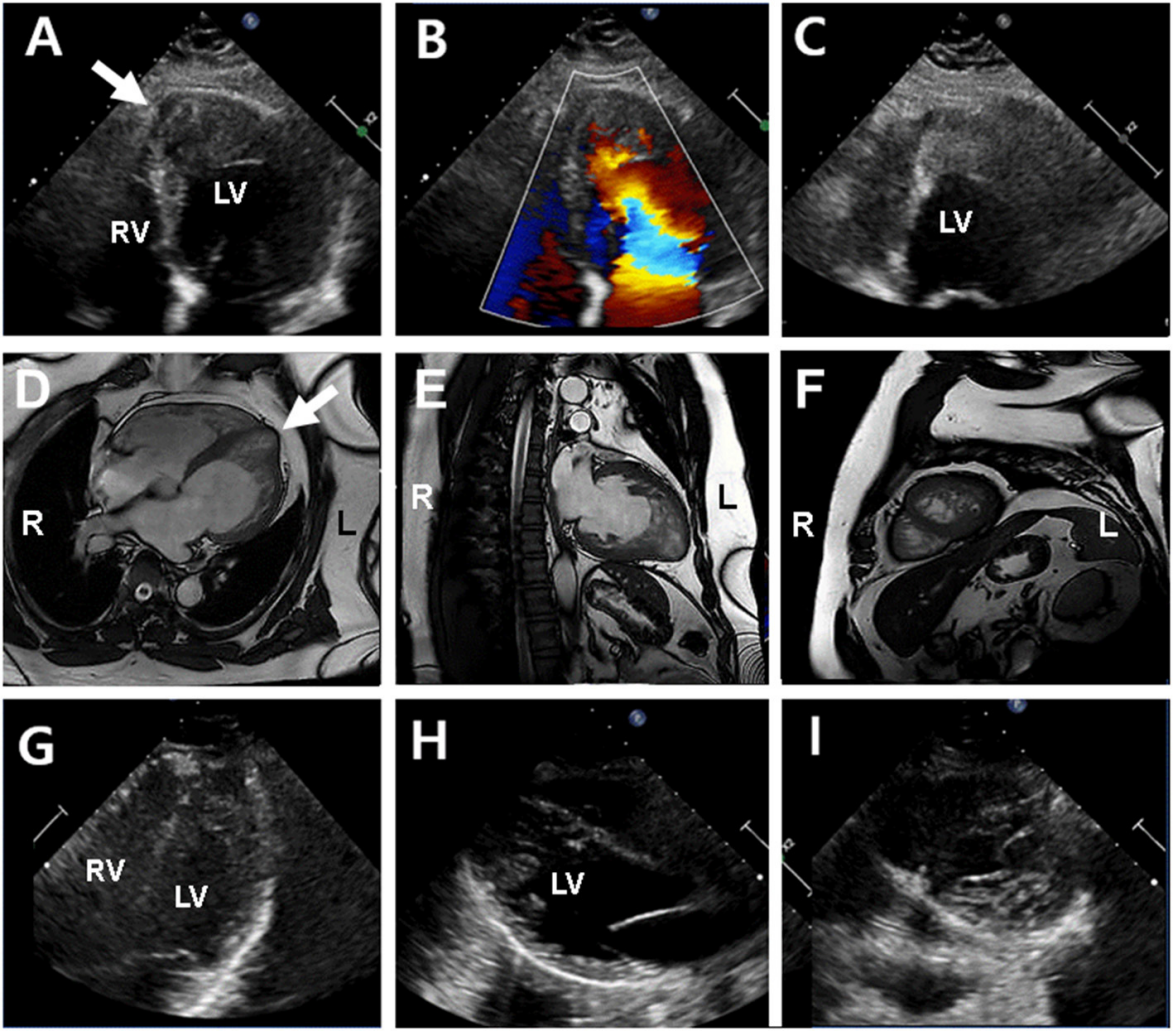
Echocardiography and cardiac magnetic resonance imaging (cMRI) in the proband's father (II-1) (upper and middle panel) and sister (III-1) (lower panel). **(A–C)** Echocardiography showed left ventricular hypertrophy and apical echogenic mass or dense trabeculation and apical septal aneurysm (arrow) in the proband's father. **(D–F)** cMRI determined dense trabeculation and focal apical septal aneurysm that considered as hypertrophic LVNC in the proband's father. **(G–I)** Echocardiography in proband's sister showed normal left ventricular function and geometry except for prominent trabeculation of left ventricle that diagnosed with isolated LVNC in the proband's sister.

A 15-year-old boy (III-2 in Figure 1A) was admitted to the Department of Cardiology, Daejeon St. Mary's Hospital (Daejeon, Korea) for the evaluation of exertional dyspnea for 2 weeks. He used to practice fencing and complained of relatively mild exertional discomfort for several years. He did not have any other metabolic or clinical abnormality except for obesity (body mass index: 31.8 Kg/m^2^). The proband underwent transthoracic echocardiography (TTE), which showed a non-compacted myocardium with a dilated cardiomyopathy phenotype such as left ventricular end-diastolic dimension (LVEDD) 60 mm, interventricular septal dimension (IVS) 8.2 mm, left ventricular ejection fraction (LVEF) 25% (Figures 2A–C). Cardiac magnetic resonance imaging (cMRI) revealed LVNC with dilated cardiomyopathy (Figures 2D–F). His electrocardiogram (ECG) and Holter monitoring showed sinus arrhythmia and non-specific intraventricular conduction delay, as well as rare atrial premature contraction (Figure 2G). He did not complain of any skeletal muscle symptoms, and serum muscle enzyme levels were normal. As previously mentioned, LVNC has been classified by the American Heart Association (14) as a primary cardiomyopathy of genetic origin, presenting in patients with marked clinical heterogeneity. As such, clinical screening of the proband's family for an inherited cardiomyopathy became necessary. The proband's 48-year-old father (II-1 in Figure 1A) had a medical history of diabetes and hypertensive heart disease. He was admitted to another hospital 2 months previously because of pleural effusion and congestive heart failure. At that time, his serum NT-proBNP level was elevated (532 pg/mL). His TTE showed an echogenic mass or trabeculation and a LVEF of 35% with aneurysmal changes in the apical wall (Figures 3A–C). A coronary artery angiogram was performed to evaluate the apical aneurysm, and it demonstrated no coronary artery stenosis. In addition, cMRI revealed dense hypertrabeculation with focal aneurysmal changes in the apical septal wall. These findings could be considered as the hypertrophic phenotype of LVNC (Figures 3D–F). The proband's 18-year-old sister (III-1 in Figure 1A) did not have any symptoms; however, her TTE revealed LVNC with normal LVEF, which was consistent with isolated LVNC (Figures 3G–I). All affected individuals did not present with skeletal muscle myopathies. The clinical characteristics of affected individuals diagnosed with LVNC are described in Table 1.

**Table 1:** Clinical characteristics of individuals harboring novel *ACTN2* variant diagnosed as left ventricular non-compaction.

**Characteristics**	**Father (II−1)**	**Sister (III−1)**	**Proband (III−2)**
Sex/Age (year)	Male/48	Female/18	Male/15
MOGE(S) nomenclature	M_LVNC+H_O_H_G_AD_E_G−ACTN2[p.Leu223Pro]_	M_LVNC_O_H_G_AD_E_G−ACTN2[p.Leu223Pro]_	M_LVNC+D_O_H_G_AD_E_G−ACTN2[p.Leu223Pro]_
Electrocardiogram and Holter monitoring	•Sinus rhythm •Non-specific intraventricular conduction delay	•Sinus rhythm	•Sinus arrhythmia •Non-specific intraventricular conduction delay •Rare atrial premature contraction
Transthoracic echocardiography	•Hypertrophic LVNC •LVEDD 55 mm •IVS 12 mm •EF 35%	•Isolated LVNC •LVEDD 35 mm •IVS 7 mm •EF 55%	•Dilated LVNC •LVEDD 60.7 mm •IVS 8.2 mm •EF 25%

*MOGE, morphofunctional characteristic (M), organ involvement (O), genetic or familial inheritance pattern (G), and an explicit etiological annotation (E); LVNC, left ventricular noncompaction; LVEDD, left ventricular end-diastolic dimension; IVS, interventricular septal dimension; EF, ejection fraction*.

The proband's grandfather (I-1 in Figure 1A) died at the age of 35 years due to gastric cancer, and his cardiac history was unknown. The proband's 74-year-old grandmother (I-2 in Figure 1A) did not have any cardiac disease except for hypertension. She did not present with any cardiac symptoms, and her TTE showed normal cardiac function and geometry. Thus, the inheritance of the proband's father was not determined presumptively, whether inherited or sporadic. The father's brother (II-3 in Figure 1A) had no clinical symptoms, lived in another area, and did not agree to be tested. The proband's mother was completely normal through birth to adolescent and showed no skeletal muscle myopathies or cardiac symptoms and was not investigated.

## Exome Sequencing of the Father–Son Duo

The study protocol was approved by the Institutional Review Board of the Catholic University of Korea. Written informed consent was obtained from the legal guardian/next of kin of the individual(s) and minor(s) involved in the study for the publication of any potentially identifiable images or data included in this article. The exomic DNA of the proband and his father was enriched using Agilent's SureSelect XT Human All Exon v5 (Agilent Technologies, Santa Clara, CA, USA). Paired-end sequencing was conducted on the Illumina HiSeq2500 (Illumina, San Diego, CA, USA) for detection of the variant, given the suspicion of familial disease at the Green Cross Genome (Yongin, Korea). Base calling, alignment, variant calling, annotation, and quality control reporting were performed using a GATK Best Practices workflow for germline short variant discovery and were manually reviewed by medical laboratory geneticists. Variants that pass the filtering criteria are as follows: Phred quality score >20, no Fisher strand bias, read depth >30 ×, allele frequency <0.1%, non-synonymous substitution or indel occurred in coding region and exon–intron boundaries, heterozygous variant in both father and son in an autosomal dominant manner, and reported to be associated with cardiac disease in the OMIM database. In particular, annotation of the identified variants with respect to the results of their variant on the reported genes and the variant effect predicted by *in silico* computational tools, such as MutationAssessor (http://mutationassessor.org/r3/) (15), Sorting Intolerant from Tolerant (https://sift.bii.a-star.edu.sg/) (16), Polyphen2 (http://genetics.bwh.harvard.edu/pph2/) (17), Likelihood Ratio Test (http://www.genetics.wustl.edu/jflab/lrt_query.html) (18), and MutationTaster (http://www.mutationtaster.org/) (19), and public genome databases from gnomAD (https://gnomad.broadinstitute.org/) and KRGDB (http://coda.nih.go.kr/coda/KRGDB/index.jsp), were estimated using the dbNSFP 2.4 (20) and Ensembl Variant Effect Predictor (21).

## Results

By estimating sequence quality along all sequences, 6,119 and 5,241 million reads were generated from the proband and his father's samples, respectively. The % bases above average 30 × were achieved for 93.6 and 91% of the target region, and the mean read depth (×) was 123 and 104, respectively. ES of the father–son duo identified eight heterozygous variants as candidate causes of autosomal dominant inherited diseases (Supplementary Table 1). Because there was no phenotypic description related to LVNC in the OMIM database, except for the *ACTN2* variant (chr1:236894585T/C), the *ACTN2* variant seems to be pathogenic. In the proband and his father presenting with a similar phenotype, the missense *ACTN2* variant causing a codon change of leucine to proline at position 223 (NM_001103.3:c.668T>C, p.Leu223Pro; no rsID) was newly identified. To confirm the presence of the *ACTN2* c.668T>C variation in the affected members, Sanger sequencing was performed. It confirmed the heterozygous c.668T>C variant in the proband, his father, and sister, while it was absent from the blood DNA of the proband's grandmother (Figure 1B). Thus, the variant identified from the affected individuals demonstrated to cause an autosomal dominant cardiomyopathy. In addition, this missense variant of the *ACTN2* was predicted to be “deleterious,” “damaging,” or “disease causing” by computational *in silico* prediction. Cross-species sequence comparisons (phastCons, SiPhy, and GERP) of amino acid sequences of ACTN2 protein revealed that this mutated site was highly conserved in vertebrates (phastCons 1 > cutoff of 0.8, SiPhy 14.464 > 12.17, and GERP 4.68 > 4.4) (Figure 1C). The variant is not listed in gnomAD or KRGDB. Paternity and kinship analysis was conducted using short tandem repeat (STR) multiplex assay (AmpFLSTR® Identifiler; Applied Biosystems, Foster City, CA, USA), and STR analysis confirmed the biological association of the parents and sibling with the proband.

## Discussion

LVNC is classified into the following subtypes: hypertrophic, hypertrophic dilated, dilated, restrictive, isolated, and biventricular (22). Our cases were consistent with hypertrophic LVNC (II-1), dilated LVNC (III-2), and isolated LVNC (III-1) in a single family, which are heterogeneous phenotypes of LVNC. It is essential to accurately assess the phenotype of LVNC because different phenotypes are associated with variable outcomes and necessitate different surveillance strategies (23). Routinely, the diagnosis of LVNC relies on non-invasive imaging studies, such as TTE and cMRI. The diagnostic criteria for LVNC are based on the ratio of thickness of the non-compacted layer to that of the compacted layer, with a ratio >2–3:1 at the end of the typical diastole (22, 24). TTE is the most common diagnostic modality because of its availability. However, cMRI can delineate the extent of LVNC more precisely and also provide additional morphological characterization of the myocardium (23, 25).

Diverse clinical phenotypes were observed in this family, including hypertrophic, dilated, and isolated LVNC. In particular, it was difficult to distinguish the dense myocardial trabeculation from LV thrombus in the proband's father because he had focal aneurysmal changes at the LV apex. In this case, cMRI could provide a high degree of spatial resolution of the LV apex. In addition to imaging studies, genetic diagnosis can be useful in elucidating the underlying genetic cause of the disease, and it allows for predictive testing of other asymptomatic at-risk members in families with such variable clinical presentations. Our cases were the first Korean cases of familial LVNC caused by a novel *ACTN2* missense variant and were identified by a duo ES approach to examine the genome of the proband and his father. As with the universal TNM staging for tumors, MOGE(S) nomenclature is a descriptive nosology that combines morphofunctional traits and organ/system involvement with familial inheritance patterns, identified genetic defects, or other etiologies (26). The MOGE(S) nomenclature for this family is shown in Table 1. The MOGE(S) system distinguishes LVNC from LV dilation and dysfunction (M_LVNC+D_) or LV hypertrophy (M_LVNC+H_) from isolated LVNC (M_LVNC_).

The *ACTN2* mutation, as a sarcomere mutation, has rarely been reported among families with LVNC (12, 27–29). The *ACTN2* encodes alpha-actinin2, which is the only muscle isoform of α-actinin expressed in the cardiac muscle. ACTN2 plays important roles, such as a structure anchor and stretch sensor, and is involved in ion channel organization in the Z-disc of cardiomyocytes (12). Moreover, ACTN2 interacts directly with the cardiac, potassium, and sodium ion channels. Molecular coupling of a Ca^2+^-activated K^+^ channel to L-type Ca^2+^ channels via alpha-actinin2 has been reported (30). Previous studies reported that the *ACTN2* mutation may lead to diverse cardiomyopathy, including dilated cardiomyopathy, hypertrophic cardiomyopathy, restrictive cardiomyopathy, and LVNC, as well as arrhythmia, such as idiopathic ventricular fibrillation, juvenile atrial arrhythmias, and sudden unexplained death (28, 31–34). Notably, the variant *ACTN2* c.683T>C (p.Met228Thr) was described by Girolami et al. (28) in a family with familial hypertrophic cardiomyopathy and juvenile atrial arrhythmias. In this study, the identified variant *ACTN2* c.668T>C (p.Leu223Pro) is next to this variant (p.Met228Thr), and ECG and Holter monitoring of the juvenile proband revealed non-specific intraventricular conduction delay, sinus arrhythmia, and premature atrial contraction. On the other hand, Alpha-actinin-2 encoded by the *ACTN2* is highly abundant in cardiac and skeletal muscle, where it plays several functional and structural roles in the sarcomeres (34). Thereby underlies the diverging pathomechanisms resulting in not only cardiomyopathy but also skeletal myopathy (35). Recently, Savarese et al. (36) have described mutations in the *ACTN2* gene causing a late onset distal myopathy. Thus, we cannot exclude the occurrence of a late onset skeletal myopathy someday in our affected individuals with an *ACTN2*-related cardiomyopathy.

Even within families with LVNC harboring the same mutation, adverse outcomes are variable (12, 28). Despite the low genotype–phenotype correlation in cardiomyopathies and cardiac channelopathies, recent guidelines and experts recommend family screening. Therefore, family screening for patients with LVNC associated with *ACTN2* mutation should be performed for early detection of the LVNC phenotype with poor outcomes, such as dilated LVNC (22, 37). While diverse types of LV morphology have also been identified in patients with mutations in *ACTN2* (28, 31, 32), this is exemplified by our study on the structural pathologies caused by Leu223Pro in the ACTN2 actin binding domain (ABD). This comprises two calponin homology domains connected via a flexible linker, which allows for conformational flexibility in the ABDs, to a rod domain on ACTN2 (11). For example, Gly111Val and Ala119Thr on the CH1 domain have small effects on the structure, function, and behavior and might contribute to a mild phenotype for this disease. In addition, digenic inheritance and genetic modifiers exert a major effect and can explain the pleiotropic phenotype (38): two genes in *ACTN2* and *CMYA5* contribute to the development of cardiomyopathy (39).

In conclusion, we described a Korean family with a novel likely pathogenic *ACTN2* variant, with LVNC manifesting as distinct heterogeneous phenotypes, such as hypertrophic, dilated, and isolated phenotypes, within the same family. Genetic screening of the family for congenital cardiomyopathy is necessary for patients with LVNC associated with *ACTN2* mutation, even if the patient has isolated LVNC that is expected to have a clinically good prognosis.

## Data Availability Statement

The original contributions presented in the study are included in the article/Supplementary Material, further inquiries can be directed to the corresponding author.

## Ethics Statement

The study protocol was approved by the Institutional Review Board of the Catholic University of Korea. Written informed consent to participate in this study was provided by the participants' legal guardian/next of kin. Written informed consent was obtained from the individual(s), and minor(s)' legal guardian/next of kin, for the publication of any potentially identifiable images or data included in this article.

## Author Contributions

JP and YC made substantial contributions to the analysis and interpretation of the NGS data and were involved in drafting the manuscript. HP made substantial contributions to the interpretations of clinical medical records. JC was involved in drafting the manuscript and in critically revising important intellectual content. All authors have read and approved the manuscript for submission, contributed to the article, and approved the submitted version.

## Conflict of Interest

The authors declare that the research was conducted in the absence of any commercial or financial relationships that could be construed as a potential conflict of interest.
