# Supplementary material for: Case Report: Novel Likely Pathogenic ACTN2 Variant Causing Heterogeneous Phenotype in a Korean Family With Left Ventricular Non-compaction
Source: Front Pediatr. 2021 Mar 30;9:609389. doi: 10.3389/fped.2021.609389 (PMC8042379; doi:10.3389/fped.2021.609389)
Supplement: Supplementary file 1 [file Table_1.docx]

| **Supplementary Table S1. Eight heterozygous variants as a candidate cause of autosomal dominant inherited diseases by father-son duo using exome sequencing** | | | | | | | | | | | | | |  |
| --- | --- | --- | --- | --- | --- | --- | --- | --- | --- | --- | --- | --- | --- | --- |
| **Gene** | **Location** | **Nucleotide ID** | **Base change** | **Codon change** | **rsID** | **OMIM** | **gnomAD** | **KRGDB** | **SIFT** | **Polyphen2** | **LRT** | **MuT** | **MuA** | |
| *ACTN2* | chr1: 236894585 | NM_001103.3 | c.668T>C | p.Leu223Pro | na | # 612158,  # 618655 | 0 | 0 | 0 | 1  (D) | 0  (D) | 1 | 4.305  (H) | |
| *CAMTA1* | chr5: 76330331 | NM_015215.3 | c.2542G>A | p.Val848Ile | rs142045456 | # 614756 | 0.0001285 | 0.003636 | 0.076 | 0.999  (D) | 0  (D) | 1 | 1.15  (L) | |
| *KRT9* | chr17: 39725708 | NM_000226.3 | c.1014A>T | p.Arg338Ser | rs753715101 | # 144200 | 0.0000358 | 0.005455 | 0 | 0.997  (D) | na | 1 | 3.1  (M) | |
| *PACS2* | chr14: 105850758 | NM_001100913.2 | c.1849G>T | p.Ala617Ser | rs781901106 | # 618067 | 0.000004018 | 0 | 0.457 | 0.037  (B) | 0.001  (N) | 1 | 0.835  (L) | |
| *PHF21A* | chr11: 45959853 | NM_016621.3 | c.1322A>G | p.His441Arg | na | # 618725 | 0 | 0 | 0.001 | 0.979  (D) | 0  (D) | 1 | 0.695  (N) | |
| *SLC25A11* | chr17: 4841515 | NM_003562.4 | c.671G>A | p.Cys224Tyr | rs201961261 | # 618464 | 0.0001957 | 0.000455 | 0.306 | 0.005  (B) | 0  (D) | 1 | 1.445  (L) | |
| *TRRAP* | chr7: 98513416 | NM_003496.3 | c.2270T>A | p.Phe757Tyr | na | # 618454 | 0 | 0 | 0 | 1  (D) | 0  (D) | 1 | 2.015  (M) | |
| *ZFHX4* | chr8: 77764208 | NM_024721.4 | c.5051C>T | p.Ala1684Val | na | # 178300 | 0 | 0 | 0.038 | 0.002  (B) | 0.073  (U) | 1 | 0.895  (L) | |
| chr, chromosome; rsID, Reference SNP cluster ID; OMIM, Online Mendelian Inheritance in Man; gnomAD, The Genome Aggregation Database v.2.1.1 exomes; KRGDB, Korean Reference Genome DB (1100 individuals: The 2nd phase); MuT, MutationTaster;, MutationAssessor; D, damaging; B, benign; N, neutral; U, uncertain; H, high; M, medium; L, low; na, not available | | | | | | | | | | | | | |  |
